# Anti-tumoral Effects of Recombinant *Lactococcus lactis* Strain Secreting IL-17A Cytokine

**DOI:** 10.3389/fmicb.2018.03355

**Published:** 2019-01-23

**Authors:** Elsa Jacouton, Edgar Torres Maravilla, Anne-Sophie Boucard, Nicolas Pouderous, Ana Paula Pessoa Vilela, Isabelle Naas, Florian Chain, Vasco Azevedo, Philippe Langella, Luis G. Bermúdez-Humarán

**Affiliations:** ^1^Micalis Institute, AgroParisTech, INRA, Université Paris-Saclay, Jouy-en-Josas, France; ^2^Instituto de Ciências Biológicas, Federal University of Minas Gerais, Belo Horizonte, Brazil

**Keywords:** *Lactococcus lactis*, lactic acid bacteria, IL-17A, cancer, HPV

## Abstract

Interleukin-17A (IL-17A) is a pro-inflammatory cytokine produced by T_H17_ cells that participates and contributes in host defense and autoimmune disease. We have recently reported antitumor properties of the probiotic strain of *Lactobacillus casei* BL23 in mice and T_H17_ cells was shown to play an important role in this beneficial effect. In order to better understand the role of IL-17A in cancer, we constructed a recombinant strain of *Lactococcus lactis* producing this cytokine and we determined its biological activity in: (i) a bioassay test for the induction of IL-6 production by murine fibroblasts 3T3 L1 cells line and (ii) in a mouse allograft model of human papilloma virus (HPV)-induced cancer. Our data show that recombinant *L. lactis* produces and efficiently secretes biologically active IL-17A cytokine. Interestingly, ∼26% of mice intranasally treated with *L. lactis*-IL-17A and challenged with TC-1 cells remained tumor free over the experiment, in contrast to control mice treated with the wild type strain of *L. lactis* which developed 100% of aggressive tumors. In addition, the median size of the ∼74% tumor-bearing mice treated with recombinant *L. lactis*-IL-17A, was significantly lower than mice treated with *L. lactis*-wt. Altogether, our results demonstrate that intranasal administration with *L. lactis* secreting IL-17A results in a partial protection against TC-1-induced tumors in mice, confirming antitumor effects of this cytokine in our cancer model.

## Introduction

Cancer remains a serious health concern in human society worldwide and colorectal cancer (CRC), prostrate, lung, stomach, liver and breast cancers are among the major types associated with significant mortality every year (Ferlay et al., 2012). Cancer is generally considered to be a disease involving both host genetics and environmental factors; however microorganisms (such as viruses and bacteria) are associated in ∼20% of human cancers (de Martel et al., 2012). Recent studies suggest that probiotics can help to fight cancer. Probiotics are live microorganism which, when administered in adequate amounts confer a health benefit on the host (Food and Agriculture Organization, 2002). For instance, probiotics can induce dendritic cells (DC) maturation (Delcenserie et al., 2008), enhance natural killer (NK) cells cytotoxicity (Takagi et al., 2001), and upregulate cytokine secretion (Delcenserie et al., 2008; Azcarate-Peril et al., 2011). It has also been reported that some strains of *Lactobacillus* can induce DC maturation and T_H1_ (antiviral and bacterial immunity) and T_H17_ (inflammation and auto-immunity) differentiation (Kemgang et al., 2014; Cai et al., 2016; Lee et al., 2016). However, despite the great number of studies that have demonstrated anti-cancer effects of different strains of *Lactobacillus* (Khazaie et al., 2012; Konishi et al., 2016; Lenoir et al., 2016), the precise host molecular mechanisms of these antitumor properties remain unclear. Next generation probiotics, such as *Akkermansia muciniphila* and *Faecalibacterium* genus as well as genetically modified microorganisms (GMOs) (O’Toole et al., 2017) have demonstrated beneficial effects in the context of cancer, promoting the immune checkpoints inhibitors therapy targeting the programmed cell death protein 1 (PD-1) and cytotoxic lymphocyte-associated antigen (CTLA-4). In addition, other studies support the role of *Bifidobacterium, Bacteroides*, *Faecalibacterium* and *Akkermansia* species in cancer therapy targeting the immune checkpoint blockade (CTLA-4, PD-1), showing a T cell-specific anti-tumor-induced response (Sivan et al., 2015; Vetizou et al., 2015; Gopalakrishnan et al., 2018; Routy et al., 2018).

We previously demonstrated that mucosal administration of the probiotic strain of *Lactobacillus casei* BL23 displays anti-tumor properties in three different murine models of cancer (Lenoir et al., 2016; Jacouton et al., 2017). Interestingly, we showed that this strain was able to modulate a T-cell immune response toward a T_H17_-biased immune response, accompanied by the expression of regulatory cytokines (e.g., IL-6, IL-17, IL-10, and TGF-β), in a murine model of CRC (Lenoir et al., 2016). In particular we were intrigued by IL-17 induction, since IL-17 seems to be essential for both metastasis and elimination of tumor cells (Murugaiyan and Saha, 2009). Thus, IL-17-producing T_H17_ cells have recently gained considerable importance in cancer (Maniati et al., 2010). Therefore, we hypothesized that IL-17-induced by *L. casei* BL23 could play an important role in the anti-tumor effect of this probiotic strain. We thus decided to use a genetically modified strain of *Lactococcus lactis*, the model lactic acid bacterium (LAB), to produce and deliver exogenous murine IL-17 and to determine its anti-tumor effect in a mouse allograft model of human papilloma virus (HPV)-induced cancer.

## Materials and Methods

### Bacterial Strains and Growth Conditions

*L. lactis* MG1363 (Gasson, 1983) was grown in M17 medium (Difco Laboratories, England) supplemented with 0.5% glucose (GM17) and 15 μg/ml of chloramphenicol at 30°C without agitation.

### Construction of a Recombinant Strain of *L. lactis* Secreting Murine IL-17

**FIGURE 1:**
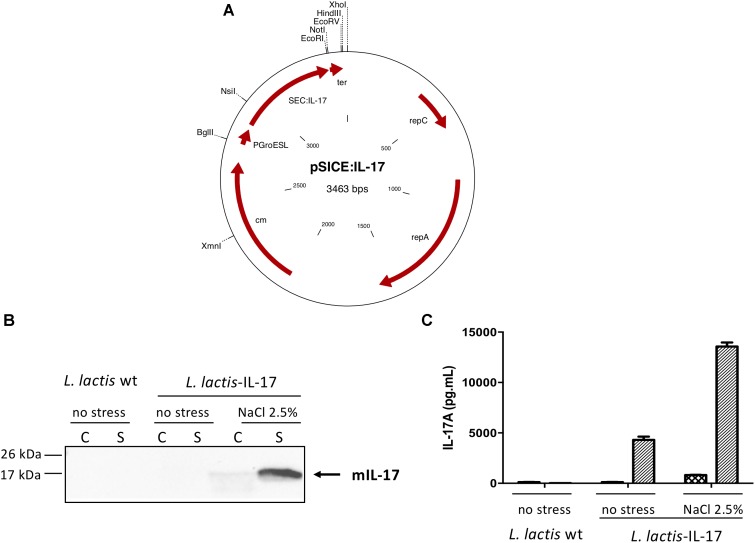
IL-17 expression by *L. lactis*. **(A)** Schematic representation of pSICE:IL-17 plasmid. Protein samples (C and S) were prepared from both non-stressed and stressed *L. lactis*-wt and *L. lactis*-IL-17 cultures and IL-17 production was assessed by **(B)** Western blot and **(C)** ELISA. Position and size of molecular mass markers is indicated on the left. The position of mature murine IL-17 is given by an arrow. Values are mean ± SEM.

To construct a vector which will allow stress-inducible IL-17 expression in *L. lactis* a DNA fragment encoding IL-17 mature sequence was obtained from a recombinant plasmid containing murine *il-17* gene (synthesized by Geneart, Invitrogen) with *Nsi*I/*Eco*RI enzymes. As previously described (Benbouziane et al., 2013), we used pLB333 vector containing *nucB* gene under the control of the stress inducible *groESL* promotor. pLB333 was digested with the same enzymes to replace *nuc* gene by *il-17* gene. The resulting vector, pSICE:IL-17 (Figure 1A), was established into *L. lactis* MG1363 strain to obtain *L. lactis*-IL-17. For detection of IL-17, *L. lactis*-IL-17 strain (*L. lactis*-*wt* was used as negative control) was grown overnight (ON, optical density (OD)_600nm_ = 2.0–2.5) as described above. Plasmid DNA isolation and general procedures for DNA manipulation were essentially performed as described previously (Sambrook et al., 1989). PCR amplification was performed using High Fidelity PCR Enzyme Mix (Fermentas) with a thermal cycler (Applied Biosystem). DNA sequences were confirmed by sequencing (MWG-Genomic Company, Germany).

### *In vitro* Validation of IL-17 Production and Secretion by Recombinant *L. lactis*

Over-night cultures were washed twice using PBS and culture pursued (after a 1:10 dilution in GM17 medium) until OD_600nm_ ∼0.6. Then, cultures were induced with 2.5% NaCl for 30 min and protein samples prepared from 2 ml of the induced cultures. After centrifugation (10 min, 17500*g*), the cellular pellet (C) and supernatant (S) were treated separately. The S samples were precipitated with 200 μl of trichloroacetic acid (TCA) 100% for 1 h on ice to recover proteins (centrifugation at 17500*g* at 4°C for 30 min) and resuspended in 200 μl of 50mM NaOH. The C fraction was resuspended in 200 μl of PBS plus protease inhibitors (Roche) and sonicated 30 s with alternated pulses on ice (on: 5 s, off: 30 s). Protein samples were diluted 1:1 in Laemmli sample buffer containing 355 mM β-mercaptoethanol and denaturated 5 min at 95°C. Equal amounts of proteins were loaded and separated on a Mini-PROTEAN TGX stain free 4–20% SDS gel at 200 V and further transferred to a PVDF membrane using a Trans-Blot Turbo transfer system (Biorad). Membrane was probed with primary antibody anti-mouse IL-17A (R&D Systems) and secondary anti-rat IgG HRP-conjugated antibody (Abliance) at 1:1000 dilutions. Bound secondary antibody was visualized by the Clarity ECL Western Substrate (Bio-Rad) and Chemidoc imaging system (Biorad). The concentration of IL-17A secreted in the medium was assessed by ELISA (mouse IL-17 ELISA Development Kit, Mabtech).

### Determination of the Biological Activity of IL-17 Produced by Recombinant *L. lactis*

Murine fibroblasts 3T3 L1 cells line, grown in DMEM medium (Lonza, Switzerland) supplemented with 10% heat-inactivated fetal calf serum (FCS), 50 U/ml penicillin and 50 U/ml streptomycin (Lonza, Levallois-Perret, France) were cultivated at 1 × 10^5^ cells per well during 24 h at 37°C, 5% CO_2._ Then, medium was changed and bacterial preparations added at 10% for supernatants, pellet and control medium or MOI 100 for bacteria suspensions during 24 h. Supernatants of co-incubations were collected and stored at -80°C before ELISA analyses (mouse IL-6 DuoSet ELISA, R&D).

### Mice and TC-1 Cell Line

Specific pathogen-free C57BL/6 mice (females, 6–8 weeks old; Janvier SAS, St. Berthevin, France) were housed in a pathogen-free isolator (*n =* 4 mice per cage) under sterile conditions in 12-h light cycles in the animal facilities of the French National Institute for Agricultural Research (INRA, IERP, Jouy-en-Josas, France). Animals were supplied with water and fed *ad libitum* (normal chow: R 03-40, SAFE). Temperature and moisture were carefully controlled. Mice were observed once a day to ensure their welfare. All protocols were carried out in accordance with the institutional ethical guidelines of the ethics committee COMETHEA (Comité d’Ethique en Expérimentation Animale of the Centre INRA of Jouy-en-Josas and AgroParisTech), which approved this study.

The mouse (C57BL/6) lung tumor cell line TC-1 (generated by transduction with a retroviral vector harboring HPV-16 E6/E7 genes plus a retrovirus expressing activated human oncogene c-Ha-*ras* (Lin et al., 1996)) was grown in RPMI medium 1640 (Lonza, Switzerland) supplemented with 10% heat-inactivated FCS, 50 U/ml penicillin, 50 U/ml streptomycin (Lonza, Levallois-Perret, France), 0.4 mg/ml G418 and 0.2 mg/ml hygromycin in 5% CO_2_ atmosphere.

### TC-1 Cell Line Challenge and Bacteria Administration

Groups of mice (*n =* 22 from 3 independent *in vivo* experiments) were intranasally (*i.n.*) administered using a micropipette with 1 × 10^9^ colony-forming units (CFU) of either *L. lactis*-wt or *L. lactis*-IL-17 strain (suspended in 10 μl of PBS). ON cultures were washed two times and finally suspended in PBS at 1 × 10^11^ CFU/ml. Each mouse received 5 μl of the solution in each nostril on days -35, -21, and -7. Control mice received identical quantities of PBS (i.e., 10 μl). Mice were challenged 7 days after the final bacterial administration (D0) by subcutaneous (*s.c.*) injection in the right rear flank with 5 × 10^4^ TC-1 cells in 100 μl of sterile PBS. The dimensions of the tumor at the site of injection were measured every week in two perpendicular directions with a caliper, and tumor volume was estimated as (length × width^2^)/2 (Bermudez-Humaran et al., 2005). Mice were sacrificed by vertebral dislocation at D28.

### Analysis of the Immune Response in Mice Treated With Recombinant *L. lactis* and Challenged With TC-1 Cells

Mice were euthanatized at D28 and spleens collected and isolated via gentle extrusion of the tissue through a 50-μm-mesh nylon cell strainer (BD). Cells were resuspended in DMEM medium supplemented with 10% FCS, 2 mM L-glutamine, 50 U/mg penicillin and 50 U/mg streptomycin. Erythrocytes were lysed with red-blood-cell lysing buffer (Sigma-Aldrich). For stimulation experiments, 1 × 10^6^ cells per well were stimulated for 48 h (37°C, 10% CO2) in DMEM medium in P24 plates in presence of PMA (phorbol 12-myristate 13-acetate) ionomycin cocktail 1× (eBioscience). Culture supernatant was frozen at -80°C until processing. Levels of the cytokines IL-6 (mouse IL-6 DuoSet ELISA, R&D), IL-17A, and IFN-γ (ELISA Development Kit, Mabtech) were determined using ELISA according manufacturer’s instructions.

### Statistical Analysis

All data are expressed as mean values and standard deviations. Data analysis was performed using the GraphPad Prism Software V.5.00. Experiments were analyzed using an unpaired *t*-test. The two-tailed unpaired Mann–Whitney test was used to evaluate differences between two groups. In all experiments, a value of *P* *<* 0.05 was considered significant (*^∗^P* *<* 0.05, *^∗∗^P* *<* 0.01, *^∗∗∗^P* *<* 0.001).

## Results

### Characterization of IL-17 Production by Recombinant *Lactococcus lactis*

Before to test the biological effect of the genetically modified strain of *L. lactis* harboring pSICE:IL-17 plasmid (Figure 1A), we first analyzed IL-17 production and secretion from both non-stressed and stressed *L. lactis*-wt and *L. lactis*-IL-17 cultures by Western blot (Figure 1B). A band of approximately 15 kDa was detected in the supernatant (S) fraction from induced cultures of *L. lactis*-IL-17 strain, which corresponds to secreted mature murine IL-17. IL-17 secretion and quantification was then determined by ELISA in C and S samples. As shown in Figure 1C, a better production of IL-17 was observed (∼3-fold) under stress conditions (i.e., NaCl 2.5%): ∼15,000 pg/ml versus ∼5000 pg/ml. As expected no IL-17 signal was detected in the negative control *L. lactis-*wt.

### Recombinant *Lactococcus lactis* Secretes a Biologically Active IL-17 Cytokine

**FIGURE 2:**
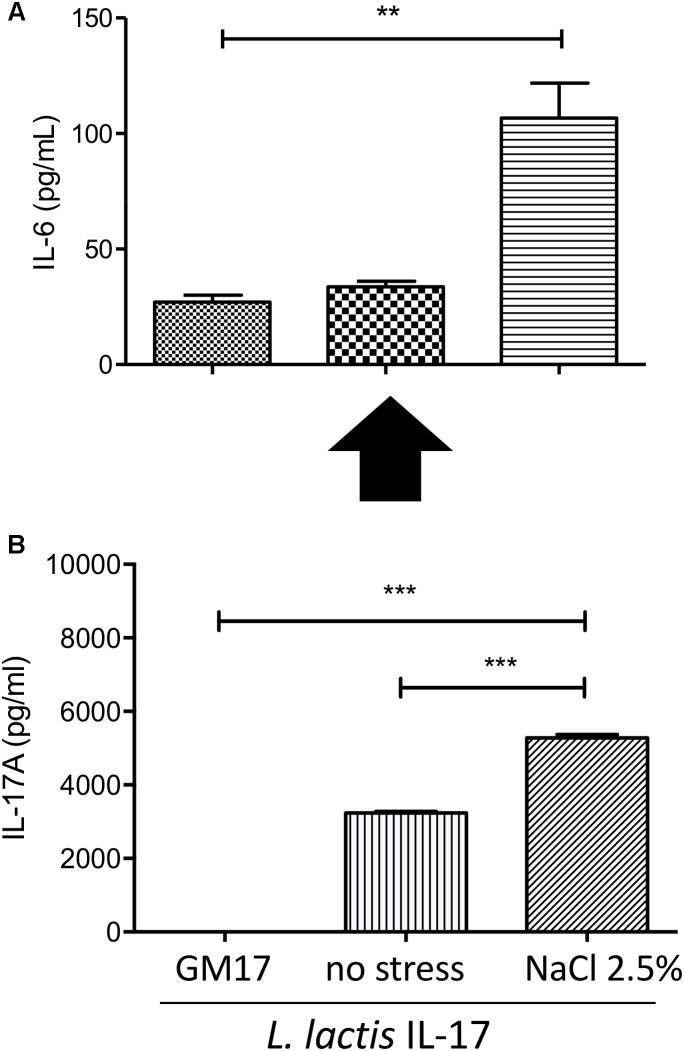
Cellular-based bioassay for *L. lactis*-secreted bioactive IL-17. **(A)** IL-6 secretion by 3T3 L1 cells after exposure to supernatant samples of recombinant *L. lactis*. **(B)** Quantification of IL-17 production by recombinant lactococci by ELISA. Data are represented as mean ± SEM of two independent *in vitro* assays. GM17 was used a control for supernatant conditions. Stress was induced with NaCl 2.5%. Data were treated by ANOVA (Turkey post-test).

Besides IL-17 detection in S samples of bacterial cultures, we determined the biological activity of this cytokine secreted by recombinant *L. lactis*. IL-17A is known to stimulate several cytokines (including IL-6) in different cell lines (such as fibroblast, epithelial cells and immune cells). Thus, we selected murine fibroblasts 3T3 L1 cells to assess specific IL-6-induction by recombinant *L. lactis*. Our results showed that S samples of *L. lactis*-IL-17 strain and stressed with NaCl 2.5% induced a significant IL-6 secretion in 3T3 L1 cells (Figure 2A) in comparison with their respective negative control. No significant IL-6 production was observed in bacterial cultures without stress induction. In parallel we confirmed by ELISA the presence of IL-17 cytokine in S samples of recombinant bacteria (Figure 2B).

### *L. lactis* IL-17 Has a Protective Effect Against Tumors in TC-1 Allograft Model of HPV-Induced Cancer

**FIGURE 3:**
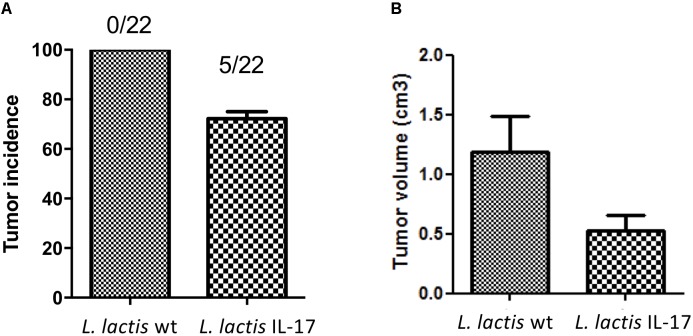
Effect of recombinant *L. lactis* expressing IL-17 against tumors in TC-1 allograft model of HPV-induced cancer. Mice were *i.n.* treated with 1 × 10^9^ CFU resuspended in 10 μl of PBS (5 μl were administered with a micropipette into each nostril) on D-35, -21, and -7. Seven days after the last administration (D0), a challenge with the tumoral cell line TC-1 was performed, and the presence and size of the tumor was monitored once a week. **(A)** Tumor incidence with proportions of tumor-free animals **(B)** Individual tumor volume at the end of week 10. Data represented mean ± SEM from 3 independent *in vivo* experiments.

To further evaluate *in vivo* the biological activity of IL-17 produced by recombinant *L. lactis,* and in particular the impact of this cytokine in the TC-1 mouse allograft model of HPV-induced cancer, we analyzed the effect after *i.n.* administration of this strain in the TC-1 tumor mice. Mice were immunized as described in Material and Methods and tumor absence/presence monitored every week. As shown in Figure 3A, *L. lactis* IL-17 displayed a protective effect against tumor development at D28 (the end of the experiment) since 77% (5/22) of mice that had been administered *L. lactis* IL-17 developed tumors with a mean tumor size of ∼0.80 cm^3^ (Figure 3B) compared to 100% (22/22) of mice receiving *L. lactis-wt* control strain (mean tumor size of ∼1.2 cm^3^) (Figures 3A,B). These results confirm that IL-17 cytokine display anti-tumor effects in our cancer model.

### *L. lactis* IL-17 Induces IL-6 and IL-17 Secretion in Reactivated Splenocytes From Mice Challenged With the Tumoral Cell Line

**FIGURE 4:**
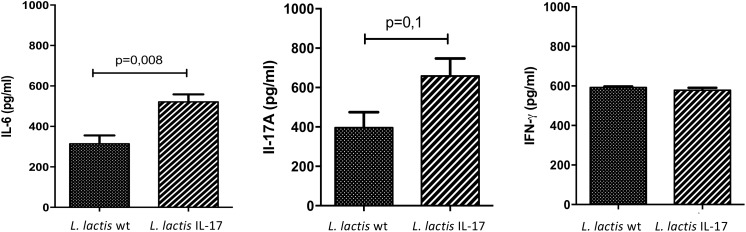
Cytokines production by reactivated splenocytes from mice treated with recombinant bacteria and challenged with TC-1 cells. Splenocytes were stimulated for 48 h with PMA ionomycin before measuring cytokines levels. Data represented mean ± SEM from 4 (*L. lactis* wt) to 8 mice (*L. lactis* IL-17). Data are analyzed with unpaired *t*-test followed by Mann–Withney post-test.

In order to further explore the impact on the immune response of mice treated with recombinant *L. lactis* IL-17 we analyzed cytokines release by reactivated splenocytes from mice 28 days after *i.n* administration of recombinant bacteria and challenged with TC-1 cells. Interestingly, *L. lactis* IL-17 induced a significant IL-6 secretion in splenocytes compared to *L. lactis*-wt (Figure 4). This modulation was correlated with a slight IL-17 induction but without reach statistical significance (Figure 4). No effect was observed on IFN-γ production by recombinant bacteria (Figure 4).

## Discussion and Conclusion

A better understanding of the interactions between cancer cells and stromal components in the tumor associated pro-inflammatory microenvironment would be important for the management of this disease (Ferlay et al., 2012). Anti-tumor response involves different components of the immune system, such as NK cells, DC, macrophages and T cells. A growing body of evidence suggests that probiotics can help to combat cancer by either protecting against gastrointestinal infections or enhancing immune response. Indeed, it has been shown that probiotics can induce DC maturation (Delcenserie et al., 2008), enhance NK cell cytotoxicity (Takagi et al., 2001), and upregulate cytokine secretion (Delcenserie et al., 2008; Azcarate-Peril et al., 2011). In addition, recent studies described the role of specific members of microbiota in cancer therapy by targeting the immune checkpoint blockade (CTLA-4, PD-1) (Sivan et al., 2015; Vetizou et al., 2015; Gopalakrishnan et al., 2018; Routy et al., 2018). Among the potential anti-tumoral mechanisms of probiotics, two of the most known are the modulation of the immune response and the induction of cellular apoptosis. For instance, two strains of *L. casei* are able to decrease tumor cell proliferation and enhance apoptosis in allograft models of CRC (Lee et al., 2004; Baldwin et al., 2010; Konishi et al., 2016). Similarly, oral administration of a *L. casei* strain reduces the onset of chemically induced tumors via the stimulation of IL-12 or NK-cell cytotoxicity mechanisms (Takagi et al., 2001, 2008). Furthermore, our team recently demonstrated protective effects of the probiotic strain *L. casei* BL23 in three different mouse models of cancer, including CRC (Lenoir et al., 2016; Jacouton et al., 2017) and the TC-1 allograft model (Lenoir et al., 2016). In one of our two CRC models (Lenoir et al., 2016), the anti-tumor effects of *L. casei* BL23 were associated with the reduction of pro-inflammatory cytokines, but the precise molecular and cellular mechanisms involved in tumor prevention of this bacterium remain unclear. Since cancer therapy includes chemotherapy, drug, vaccines, and cytokines, and for instance, current therapies are toward to enhance the immune system as use of pro-inflammatory cytokines (such as IL-2, one of the first cytokines used in cancer therapy) and immune check points inhibitors (CTLA-4, PD-1). In this work, we constructed a recombinant strain of *L. lactis* expressing IL-17. Strikingly, we showed that *i.n.* administration of this strain results in a lower tumor incidence and that tumor size was reduced in comparison to the control *L. lactis-wt*, a LAB strain for which no positive effect has been reported in the HPV-induced cancer model. Our results not only suggest a positive effect of IL-17 but also reinforce the idea that some of the molecular mechanisms of *L. casei* BL23 against cancer could be related to activation of T_H_ and NK *via* T_H17_. IL-17 is a pro-inflammatory cytokine, although its role is controversially, some studies report that IL-17 deficiency state may have a protective role or a harmful role in tumorigenesis (Welch et al., 2015; Qian et al., 2017). For example, in IL-17 deficient mice, enhanced lung and subcutaneous tumor growth and metastasis is correlated to a decrease in the number of IFN-γ producing NK cells (Kryczek et al., 2009). Recent research provided substantial insights into the mode of action of IL-17 cytokines in a variety of tumors, suggesting an anti-tumor activity of IL-17 could be achieved by means of a T cell-dependent mechanism increasing generation of specific cytotoxic T lymphocytes (Alshaker and Matalka, 2011). Paradigms are changing, and IL-17 cytokines are double-edged agents acting in a cancer-type depending manner as anti- and pro-tumor cytokines (Fabre et al., 2016). IL-6 is a pro-inflammatory cytokine involved, in part, in a TH17-related immune response with a feedback loop. Thus, we assessed the *in vivo* ability of *L. lactis* secreting IL-17 to stimulate the TH17 pathway. Here, we demonstrated an *in vivo* induction of IL-6 resulting in an increase of IL-17. We hypothesized that the anti-tumoral effect of recombinant lactococci secreting biologically active IL-17 could due to a TH17 immune response even if more experiments are needed to further decipher the precise molecular mechanisms.

In conclusion, our results demonstrate that *i.n.* administration with a genetically modified strain of *L. lactis* secreting IL-17 results in a partial protection against TC-1-induced tumors in mice, confirming antitumor effects of this cytokine in this model.

## Author Contributions

EJ and LB-H conceived and designed the study. EJ, ETM, and A-SB conducted all experiments. NP, APV, IN, FC, and VA contributed analytic tools. EJ, ETM, PL and LB-H performed the data analysis. ETM and LB-H wrote the manuscript.

## Conflict of Interest Statement

The authors declare that the research was conducted in the absence of any commercial or financial relationships that could be construed as a potential conflict of interest.
